# A malaria outbreak in Naxalbari, Darjeeling district, West Bengal, India, 2005: weaknesses in disease control, important risk factors

**DOI:** 10.1186/1475-2875-8-288

**Published:** 2009-12-10

**Authors:** Puran K Sharma, Ramakrishnan Ramanchandran, Yvan J Hutin, Raju Sharma, Mohan D Gupte

**Affiliations:** 1 Field Epidemiology Training Progamme (FETP), National Institute of Epidemiology (NIE), Indian Council of Medical Research (ICMR) R 127, Third Avenue, Tamil Nadu Housing Board, Phase I and II, Ayapakkam, Chennai, TN, 600077, India; 2 Department of Health and Family Welfare, Darjeeling district, West Bengal, India; 3 World Health Organization, India country office, New Delhi, India; 4 Postgraduate Government College, Darjeeling, West Bengal, India

## Abstract

An outbreak of malaria in Naxalbari, West Bengal, India, in 2005 was investigated to understand determinants and propose control measures. Malaria cases were slide-confirmed. Methods included calculation of annual blood examination rates (ABER, number of slides examined/population), collection of water specimens from potential vector-breeding sites, sorting of villages in categories depending on the number of abandoned wells within two kilometers radius and review of the DDT spray coverage. Cases were compared with matched neighbourhood controls in terms of personal protection using matched odds ratios (MOR). 7,303 cases and 17 deaths were reported between April 2005 and March 2006 with a peak during October rains (Attack rate: 50 per 1,000, case fatality: 0.2%). The attack rate increased according to the number of abandoned wells within 2 kilometres radius (P < 0.0001, Chi-square for trend). Abandoned wells were Anopheles breeding sites. Compared with controls, cases were more likely to sleep outdoors (MOR: 3.8) and less likely to use of mosquito nets and repellents (MOR: 0.3 and 0.1, respectively). DDT spray coverage and ABER were 39% and 3.5%, below the recommended 85% and 10%, respectively. Overall, this outbreak resulted from weaknesses in malaria control measures and a combination of factors, including vector breeding, low implementation of personal protection and weak case detection.

## Background

While malaria is mostly an endemic disease, it may also occur as outbreaks, for example in areas with low seasonal transmission [1]. Factors that may cause outbreaks include an increase in vector breeding sites, migration of infected people into a vector-rich area populated with susceptible individuals, arrival of new efficient vectors, breakdown of vector control measures, resistance of the parasites to treatment and resistance of the vectors to insecticides [2,3]. In the South-East Asia WHO region, focal outbreaks are common in Indonesia, India, Bangladesh, Myanmar, Thailand and Sri Lanka [1].

In India, the national malaria control programme is based upon (1) indoor residual spraying with DDT and other insecticides, (2) insecticide-treated nets, (3) larval control and (4) case detection and effective treatment. As per the routine, standard national programme procedures, case detection is both active and passive. For active case detection, community health workers search for cases of fever in homes every two weeks to collect blood smears. For passive case detection, health care workers in health care facilities collect blood smears among patients presenting with fever. In 2005 in India, chloroquine and primaquine were still the first-line drugs for *Plasmodium falciparum *and *Plasmodium vivax *except in designated drug-resistant areas. While the national programme struggles to control malaria in India, outbreaks remain common in various areas, including Northeastern states [4,5], Uttar Pradesh [6], and Gujarat [7].

The Indian state of West Bengal is endemic for malaria, accounting for 11% and six percent of the national malaria and *P. falciparum *caseloads, respectively (National Vector Borne Diseases Control Programme data) [8]. *Plasmodium falciparum *accounted for 32% of the state malaria cases in 2004 (Directorate of Health Services, West Bengal, India, 2004). In Darjeeling district, malaria is endemic in three community development blocks. Naxalbari (2001 population: 144,915), one of these three blocks, is located at the foothills of the Himalayas, between the 26° 38' and 26° 49' Northern latitudes and between the 88° 10' and 88° 22' Eastern longitudes, at an average elevation of 152 metres. The average maximum and minimum atmospheric temperatures are 32°C and 15°C, respectively, with an average annual rainfall of 3,000 mm. Land is used by tea estates, farming plots, forests, small streams and villages. There are a few scattered paddy fields. 17 health care facilities, few rudimentary tea garden dispensaries and a rural hospital cover the healthcare needs of the population. The area, close to Nepal, is prone to population movements. Furthermore, the proportion of chloroquine-resistant *P. falciparum *exceeded 50% in an in-vivo trial in 2003 [9]. The Naxalbari block was declared chloroquine-resistant, and second line therapy recommended. However, in August 2005, before this recommendation was ever implemented, the Naxalbari rural hospital reported an increase in the number of malaria cases. This cluster was investigated to assess its magnitude, identify its determinants and propose control measures.

## Methods

### Laboratory methods

Laboratory technicians stained and examined blood slides (thick and thin smears) as part of their routine work with a 100 × oil immersion microscope at the microscopy centre of the Naxalbari rural hospital. As per the routine quality assurance programme, all positive slides and 5 - 10% of negative slides were checked at the regional laboratory in Kolkata, the state capital.

### Descriptive epidemiology

A case of malaria was defined as an acute febrile illness with a peripheral blood smear positive for malaria or a positive rapid antigen test in a resident of Naxalbari block between April 2005 and March 2006. Malaria surveillance reports were reviewed for 2000-2005. The 2005 data was compared with the average monthly number of cases during 2000-2004 to determine whether the epidemic threshold (more than two standard deviations to the mean) had been crossed [10]. Health care facility records were searched for cases and deaths. Treatment records were reviewed for malaria deaths. Laboratory registers were reviewed to abstract slide examinations results.

Rates of malaria by age and sex were calculated using the 2001 census data as denominators. The annual blood examination rate was calculated by dividing the total number of slides examined for malaria parasites by the total population and expressed as a percentage. The slide positivity rate was calculated by dividing the total number of slides positive for malaria by the total number of slides examined and expressed as a percentage. The proportion of malaria cases detected by active detection was estimated by dividing the total number of cases detected by active detection by the total number of malaria cases and expressed as a percentage. Attack rate rates by area were represented on a map according to locations of potential breeding sites. An epidemic curve was constructed. The number of malaria cases detected through active case detection and the case fatality ratio was plotted over time to clarify the impact of increased active case detection on the reported rates (the initial reports of a cluster stimulated increased active case detection).

### Analytical epidemiology

The clustering of cases in the proximity of the abandoned wells led to suspect them as breeding sites that could have contributed to the outbreak. To test this hypothesis, the attack rate of malaria from April 2005 to March 2006 was calculated according to abandoned wells in the proximity. Villages were categorized in four levels of exposure according to the number of abandoned wells within two kilometres radius (The usual flight range of anopheles) Attack rate ratios according to the level of exposure and Chi-square for trend were calculated.

A case-control study (using the same case definition) was conducted to examine the effectiveness of personal vector protection practices in the population. Neighbours with no fever for the last three months were selected as healthy controls and matched for age and sex to malaria case-patients identified by active case detection in September 2005. A standardized questionnaire was used to collect information about selected practices, including sleeping indoors, use of repellents, use of mosquito nets and indoor residual spray with DDT. Matched odds ratio (MOR) for discordant pairs were calculated using the Epi Info 2000 software (CDC, Atlanta, GA, USA). The fractions of cases attributable to the risk factors (or the failure to use protection measures) in the population was calculated using the classical formula (the proportion of cases exposed multiplied by [odds ratio-1/odds ratio]).

### Environmental assessment

Rainfall data were reviewed from the district public health office for 2002-2005. Selected case-patients and health workers were interviewed to collect qualitative information on potential mosquitoes breeding sites. The DDT spray coverage for 2003-2005 and available information regarding drug efficacy locally was reviewed.

Water specimens were collected from potential breeding sites including ponds, rice fields, wells and slow moving streams within five kilometres radius of the malaria-affected areas. Anopheline mosquito larvae were searched using dippers of 10 centimetres diameter and 300 ml capacity (Five dips at each site). Larval densities were expressed per site as the number of larvae per five dips. Adult anopheles specimens were collected from 50 human dwellings and 25 cattle sheds in the most affected areas using the suction method and identified using morphological identification keys (without estimation of density).

## Results

### Descriptive epidemiology

The Naxalbari block reported 7,303 cases of malaria (Attack rate: 50 per 1,000, Table 1) and 17 malaria deaths (Case fatality: 0.2%, Table 1) between April 2005 and March 2006. Of the 7,303 blood smears testing positive for malaria, 4,779 (65%) had *P. vivax*, 1,517 (21%) had *P. falciparum *and 1,007 (14%) had *P. falciparum *and *P. vivax*. 3,679 (50%) cases were detected by active detection.

**Table 1:** Malaria cases and deaths by age and sex, Naxalbari, Darjeeling, West Bengal, India, April 2005 - March 2006^a^

Characteristics		Population	Cases	Deaths	Case fatality ratio (%)	Attack rate per 1,000
**Age**	0 - 4	15,216	509	0	0	33
	5 - 14	36,084	1,884	8	0.4	52
	15 - 29	42,605	2,689	3	0.1	63
	30 - 44	20,868	1,412	1	0.07	68
	45 - 59	22,317	568	3	0.5	25
	60+	7,825	241	2	0.8	31

**Sex**	Male	75,831	3,979	9	0.2	52
	Female	69,084	3,324	8	0.2	48

**Total**		144,915	7,303	17	0.2	50

^a ^Includes cases detected through active and passive case detection (See text). For a break down of the two case detection strategies, see Figure 1

While the alert was only given in August 2005, a retrospective analysis of the data indicated that the alert threshold (i.e., number of reported cases exceeding by two standard deviations the 2000-2004 average number of reported cases) had been almost reached in June 2005 (48 cases reported for a threshold of 50) and crossed in July 2005 (102 cases reported for a threshold of 57). For the whole year, the 2005 malaria caseload exceeded the 2003 and 2004 burden by a factor 10 and a factor 30, respectively. The proportion of slides that were positive (referred to as slide positivity rate in India) increased from 6.9% in 2003 and 3.9% in 2004 to 26% in 2005. On that basis, the event was determined to be an outbreak and not a seasonal increase in the number of cases.

Before the outbreak, in 2003 and 2004, the annual blood examination rates were three and four percent, respectively. It was usually 0.1% between January and May before the transmission season, increased to an average of 0.7% between June and September during transmission and reached 4.3% between October and December. In 2005, it increased to 16%, with variations throughout the year while the slide positivity rate was 26%.

**Figure 1:**
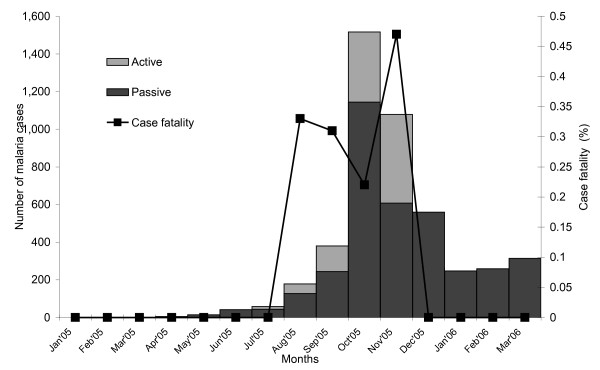
**Malaria cases by months of onset, active case detection and case fatality ratio in Naxalbari, Darjeeling, West Bengal, India, 2005 - 2006**.

**Figure 2:**
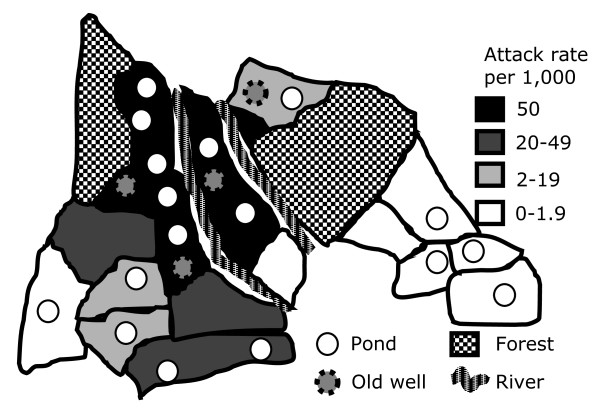
**Attack rate of malaria by sub-centres in Naxalbari, Darjeeling, West Bengal, India, April 2005 - March 2006**.

The attack rate of malaria increased from 0.3 per 1,000 in April 2005, peaked in October 2005 (18 per 1,000) and declined from November 2005 (12 per 1,000) to remain stable between January and March 2006. Incidence of malaria with *P. falciparum *and *P. vivax *peaked at the same time. The proportion of malaria cases detected through active case detection increased from 13% in June 2005 (Figure 1), reached a peak in November 2005 and decreased to 32% in December 2005. The Naxalbari block had at least one water body in each sub-centre area (the areas served by a sub-centre, the most peripheral unit in the health system). There were four abandoned wells, three of which were located in the sub-centre areas with the highest attack rate (Figure 2). The highest attack rate exceeded 60 per 1,000 and was reported among younger adults (Table 1). There was no difference between males and females.

The overall case fatality was 0.2%, with a peak at 0.5% in November 2005, highest among persons 60 years of age and older (0.8%, Table 1), with both sexes equally affected. Of 17 malaria patients who died, four had taken chloroquine and primaquine within 48 hours, 11 had taken anti-malarials ≥ 48 hours after the onset of fever and two had not taken anything. In addition, 13 (76%) were hospitalized after ≥ 48 hours of fever. Treatment received among patients who died included injected artemether (N = 11), chloroquine and primaquine (N = 4) and sulphadoxine-pyrimethamine (N = 2).

### Analytical epidemiology

The attack rate of malaria increased from 18 per 1,000 in the villages that did not have any abandoned wells within two kilometres to 824 per 1,000 among those with three abandoned wells (Table 2), with a linear trend (Chi-square for trend: 6,769; P < 0.0000, Table 2).

**Table 2:** Attack rate of malaria according to the number of abandoned wells within two kilometres, Naxalbari block, Darjeeling district, West Bengal, India, April 2005-March 2006^a^

Number of wells within 2 kilometres	# Cases	Population	Attack rate per 1,000	Ratio
**0**	636	35,063	18	Reference
**1**	1,511	25,792	59	3
**2**	4,010	22,245	180	10
**3**	552	670	824	45

**Overall**	6,709	83,770	80	-

^a ^Chi Square for linear trend = 6769; p value = < 0.0000

534 cases and 534 matched healthy neighbourhood controls were included in the case control study. Cases and controls did not differ with respect to median age (p-value = 0.9), proportion of males (p-value = 0.5). Sleeping outdoors was associated with malaria (odds ratio: 3.8, 95% confidence interval: 2.2-6.5, attributable fraction in the population: 26%, Table 3). Use of repellent and bed net was associated with lower odds of malaria (odds ratio: 0.1 and 0.3, respectively). Failure to use repellents and mosquito nets accounted for 69% and 57% of malaria cases in the population, respectively (Table 3). Indoor residual spray with DDT was uncommon among both cases and controls (40% and 41%, respectively) and was not associated with illness (Table 3).

**Table 3:** Personal protection and vector control among malaria case-patients and matched controls, Naxalbari, Darjeeling, West Bengal, India, 2005

Characteristics	Cases (n = 534)		Controls (n = 534)		Matched odds ratio	
			
	#	%	#	%	Estimate	95% confidence interval
**Sleeping outdoors every night**	190	36	150	32	3.8^a^	2.2 - 6.5
**Using coils or repellent creams daily**	120	23	152	29	0.1^b^	0.06 - 0.3
**Using bet nets every night^c^**	98	18	133	25	0.3^d^	0.1 - 0.5
**Underwent indoor residual spray (DDT)**	213	40	220	41	0.9	0.7 - 1.2

^a ^Fraction attributable to sleeping outdoors: 26%

^b ^Fraction attributable to the failure to use repellents: 69%

^c ^No insecticide impregnation in place at the time of the outbreak

^d ^Fraction attributable to the failure to sleep under a bed net: 57%

### Environmental assessment

The annual rainfall data for Naxalbari were 2,636 mm, 3,399 mm and 2,841 mm for 2002, 2003 and 2004, respectively. However, rainfall for 2005 was lower (2,230 mm). There was no recent unusual population migration for the area. The four abandoned wells had larvae of anopheline mosquitoes (average larval density: 32 per five dips). Of the 10 ponds checked for the presence of anopheline larvae, one located in the malaria-affected area was positive (larval density: four per five dips). Rice fields (n = 6), slow moving streams (n = 3) and wells in use (n = 6) did not have anopheline larvae. Of the 102 adult anopheline mosquitoes collected, 79 (77%) were *Anopheles culicifacies *and 33 (23%) were *Anopheles minimus*. Indoor residual spray coverage of households with DDT was under the 85% recommended by WHO, ranging between seven percent and 39% between 2003 and 2005.

## Discussion

Outbreaks of malaria are often complex, multi-factorial and may have natural and human-made determinants [2,10]. In 2005-2006, in Naxalbari, a multidisciplinary investigation led to suspect that a number of factors operated. These included abandoned wells where the vector bred, inadequate vector control measures, low implementation of personal protection and resistance to chloroquine. In addition, weak case detection delayed the response.

During this outbreak, identification of risk factors was challenging. Traditionally, in field epidemiology, outbreak investigations include a first step of hypothesis generation and a second step of hypothesis testing. The hypothesis generation used in this investigation process involved a study of the geographical distribution of the malaria cases among all the villages in the block. This spatial analysis pointed to the abandoned wells in the neighbourhood of the malaria-affected villages. This hypothesis could not be tested using a classical case control or cohort approach using individuals as the sampling unit. Thus, villages grouped by exposure level were compared in terms of attack rate. This comparison supported our hypothesis and pointed to the abandoned wells as breeding sites that may have contributed to this outbreak. An environmental assessment indicated that the wells contained anopheline larvae and the reported rates dropped from 11 November 2005 when the abandoned wells were closed. A similar analytic approach in Sri Lanka indicated that people living closer to established vector breeding sites were at higher risk for malaria than those living farther away [11,12]. Several studies have implicated abandoned wells as breeding sites [13,14]. *An. culicifacies *abounds in the village wells of India. It breeds in domestic wells, clean water, agricultural drains, puddles and paddy fields [15]. Effective larval control measures for abandoned wells include filling up and use of expanded polystyrene beads [16,17].

In addition to vector proliferation, low coverage of indoor residual spraying may have contributed to this outbreak. In 2003, 2004 and 2005, the DDT spray coverage was 7%, 11% and 39%, respectively. This is lower than the WHO-recommended minimal level of 85%. This low coverage would at most lead to a very modest effect on transmission [18]. The absence of association between DDT spraying and individual infections at the level is not surprising. Spraying works through a community effect. Hence, effect at individual level could only have been expected if the spraying had been highly clustered. Breakdown of vector control measures over the years progressively increases seasonal peaks and may finally culminate in an outbreak [18]. In addition, the susceptibility of *An. culicifacies *to DDT needs to be monitored as resistance has been reported [15].

Malaria incidence is influenced by the practices of the population in terms of personal protection. These were explored using a case-control study that indicated that sleeping outdoors was a risk factor while use of mosquito nets and repellents was associated with a lower risk. Repellent, including mosquito coils and creams were popular in this population who liked to sleep outdoor. The proportion of cases and controls using bed nets did not exceed 25%, in the absence of systematic activities of distribution before the outbreak. The health authorities of Naxalbari distributed 5,000 insecticide-treated bed nets among the affected population in October 2005. Further measures are needed to increase the ownership and utilization of insecticide-treated bed nets to reduce morbidity and mortality due to malaria.

WHO recommends artemesinin-based combination therapy for the treatment of patients suffering from *P. falciparum *malaria during outbreaks. Artemesinin derivatives are effective in obtaining a rapid reduction of parasitaemia. This leads to clinical recovery and a rapid reduction of gametocyte carriage [19,20]. However, during this outbreak, and until 10 October 2005, chloroquine and primaquine were the first-line drugs for all malaria patients, including those with *P. falciparum*. As the prevalence of chloroquine resistance among *P. falciparum *already exceeded 50% in Naxalbari in 2003, treatment with chloroquine and primaquine may have resulted in an increase in transmission and may also have contributed to the deaths [19,20]. On the basis of our findings, and along with the national policy, the authorities changed the treatment protocol for *P. falciparum *cases to sulphadoxine-pyrimethamine and primaquine. Within one month, the reported rates and the case fatality decreased. However, the timing of this decrease in mortality and morbidity also coincided with the change in season. Thus, it could not only be attributed to the change in treatment protocol (Figure 1).

In Naxalbari, the annual blood examination rates were under the recommended 10% threshold in 2003 and 2004. Though the overall annual blood examination rate increased to 16% in 2005, the rate remained below 0.5% from January to May, before the transmission season. This may have delayed the recognition of outbreak. Active case detection increased when malarial deaths occurred and decreased when the case fatality improved (Figure 1). This suggests that deaths triggered intensified active case detection. Thus, the annual blood examination rate exceeding 16% in 2005 was not uniform throughout the year. The breakdown of malaria case detection activities may have resulted in the build-up of a reservoir of malaria parasites in the community that might have facilitated the outbreak [19,20].

The present investigation suffered from a number of limitations with respect to the rudimentary entomological investigation. First, the species of the larvae found in the wells could not be checked. Second, a vector incrimination study with a dissection of the salivary glands of the adult mosquitoes caught could not be conducted for detection of sporozoites of the malaria parasite. Thus, *An. culicifacies *and *An. minimus *could not be conclusively identified as the vectors involved in the outbreak. Of these two vector species, *An. minimus *is usually the more efficient, but it is less likely to breed in wells than *An. culicifacies *[21]. Third, a lower, but more spread out larval density in the paddy fields could have been missed. However, the paddy fields were scattered throughout Naxalbari, hence, their involvement as breeding sites would not had led to the spatial clustering observed. Overall, these two anopheles species were the only ones identified in the area and previous incrimination studies pointed to both species as predominant vectors in Naxalbari and the Himalayan foothill regions [9,22].

The malaria outbreak in Naxalbari in 2005 was multi-factorial. Determinants included vector breeding in abandoned wells, breakdown in vector control, low implementation of personal protection, chloroquine resistant *P. falciparum *and weak case detection. On the basis of these conclusions, a number of recommendations were formulated. First, all potential vector breeding sites must be identified for implementation of larval control measures. Abandoned/unused wells need to be filled up while EPS beads. Larvivorous fishes may be used in those to be kept for future use. Second, vector control measures need to be scaled up to reach the recommended threshold. Third, the population needs to protect itself using insecticide-impregnated mosquito nets. Those need to be introduced on a broader scale, using financial assistance for those under the poverty line and promoted with messages to ensure proper use. Fourth, weekly rather than monthly reporting intervals must be used to detect outbreaks early through the calculation of weekly epidemic thresholds on the basis of historical data [10]. This method was already introduced in a neighbouring block of the same district [23]. Fourth, though sulphadoxine-pyrimethamine has already been introduced, a shift must occur towards the combination of artesunate and sulphadoxine-pyrimethamine for *P. falciparum *patients as the first-line drug, as recommended by WHO on the basis of widespread reports of resistance to sulphadoxine-pyrimethamine [19,20]. The recent revisions of the national programme are actually consistent with that orientation and propose to increase the use of artesunate combination in the area. To follow up on this outbreak and to learn all lessons, our team evaluated the National Malaria Control Programme locally in 2006. The results of this evaluation along with the conclusions of this investigation provided policy guidance that should facilitate effective prevention and control of malaria in the region in the coming years. Finally, the current plans of the Government of India to increase the availability of entomologists to conduct quality investigations of vector-borne disease outbreaks in the field should address in the future some of the limitations of our entomological investigation.

## Competing interests

The authors declare that they have no competing interests.

## Authors' contributions

MDG, PKS and SR conceived the investigation. PKS designed the protocol with SR; SR and PKS conducted the entomological study; PKS conducted analysis and interpretation of the data under the supervision of RR, YJH and MDG under the Indian Field Epidemiology Training Programme (FETP). PKS and YJH drafted the manuscript. All authors read and approved the final manuscript. PKS and RR are guarantors of the paper.
